# Age-indexed cumulative risk of high myopia estimated from axial length and early axial elongation: a 7-year school-based longitudinal cohort study

**DOI:** 10.3389/fmed.2026.1939322

**Published:** 2026-10-05

**Authors:** Jing Yang, Hui Li, Bei Du, Desheng Song, Nan Jin, Ruihua Wei

**Affiliations:** Tianjin Key Laboratory of Retinal Functions and Diseases, Tianjin Branch of National Clinical Research Center for Ocular Disease, Eye Institute and School of Optometry, Tianjin Medical University Eye Hospital, Tianjin, China

**Keywords:** axial elongation, axial length, clinical prediction model, discrete-time survival analysis, high myopia, risk prediction

## Abstract

**Purpose:**

To develop and validate age-indexed models estimating the cumulative risk of high myopia, defined as axial length (AL) ≥ 26.0 mm, using routinely collected ocular biometry, and to evaluate their potential for clinical risk stratification.

**Methods:**

This 7-year school-based longitudinal cohort included 7,052 Chinese children aged 6–16 years at baseline who participated in a school-based screening program in Tianjin, China, from 2018 to 2024. Discrete-time survival models with age as the timescale estimated cumulative risk from ages 9 to 18 years. Two models were tested: baseline AL plus sex and baseline AL plus first-year axial elongation (ΔAL) plus sex. Performance was assessed in a participant-level temporal holdout test set using iAUC, iBrier, calibration, and decision-curve analysis, and by school-level internal-external cross-validation using iAUC and iBrier.

**Results:**

Overall, 577 participants (8.2%) developed high myopia. In the temporal holdout test set (*n* = 5,245), the AL + ΔAL model outperformed the AL-only model, with higher discrimination [iAUC, 0.919 [95% CI, 0.902–0.933] vs. 0.851 [95% CI, 0.833–0.869]] and lower prediction error [iBrier, 0.074 [95% CI, 0.068–0.081] vs. 0.087 [95% CI, 0.080–0.095]]. Calibration was close to ideal, decision-curve analysis showed clinical net benefit, and school-level validation confirmed stable performance.

**Conclusions:**

Routine AL measurement, particularly when combined with early axial elongation, enables accurate age-specific risk estimation of AL-defined high myopia and provides a transparent basis for timely and proportionate myopia-control decisions.

## Introduction

Myopia is a major global public health concern, particularly among children and adolescents. Recent estimates indicate that the prevalence of myopia in this demographic has risen from 24.32% in 1990 to 35.81% in 2023, with projections reaching 39.80% by 2050, affecting over 740 million individuals worldwide (1). For the overall population, approximately 50% are projected to be myopic and about 10% to have high myopia by 2050 (2). High myopia is associated with sight-threatening complications, including myopic macular degeneration, retinal detachment, glaucoma, and cataract, highlighting the need for early identification and effective control (3–5).

In clinical and epidemiologic settings, high myopia is commonly defined as spherical equivalent ≤−6.00 diopters (D) or axial length (AL) ≥ 26.0 mm. Although spherical equivalent ≤−6.00 D and AL ≥26.0 mm are often regarded as corresponding clinical thresholds, they are not equivalent at the individual level because ocular biometric components vary across individuals (6). Importantly, the risk of pathologic sequelae is more strongly linked to axial elongation than to refraction (7–10). Together with consensus statements that emphasize axial length as the principal structural metric in high myopia, these observations support focusing on axial growth as the key target for prevention and early management (11).

A range of interventions can slow childhood myopia progression, including increasing outdoor time, low-dose atropine, optical strategies such as defocus-incorporated multiple segments spectacle lenses, dual-focus soft contact lenses, orthokeratology, repeated low-level red-light therapy, and selected combination regimens (12–18). Short-term atropine exposure has also been associated with measurable changes in ciliary muscle and choroidal parameters in children (19). However, candidacy and expected benefit vary across children, and each option carries monitoring requirements, costs, and potential adverse effects (20–22). Estimating an individual's cumulative risk of high myopia may support risk-adapted follow-up and inform discussions regarding myopia-control strategies.

Artificial intelligence (AI) and machine-learning methods are increasingly being applied to ophthalmic disease diagnosis and risk prediction, reflecting a broader shift toward data-driven clinical decision support (23–25). Bibliometric analyses have also documented evolving hotspots and trends in global myopia research (26). Within myopia research, a growing body of work predicts who will develop high myopia using diverse methods and inputs, including environmental and genetic factors and ocular characteristics. However, most studies define the endpoint by refraction (spherical equivalent ≤−6.00 D) and rely on measures that are not readily obtainable in routine clinics or school screening (e.g., cycloplegic refraction, imaging, or genotyping) (27–31). Therefore, prediction models based on routinely measured ocular biometry may be useful for clinical and school-based risk stratification.

This longitudinal, school-based study presents pragmatic models based on routinely available biometric and demographic variables that estimate a child's cumulative risk of high myopia (AL ≥26.0 mm) from age 9 to 18 using baseline AL, 1-year axial elongation (ΔAL), sex, and age. Because these measures are routinely collected, the models are straightforward to implement in outpatient care and school screening, enabling early identification and prioritization of candidates for myopia-control interventions.

## Methods

### Study population

This 7-year, school-based longitudinal cohort was conducted through annual vision screenings at 7 primary and secondary schools in Tianjin, China, from 2018 to 2024. The study was approved by the Institutional Ethics Committee of Tianjin Medical University Eye Hospital (HSEARS20230210006). All procedures adhered to the Declaration of Helsinki. Written informed consent was obtained from parents or legal guardians before screening. Race and ethnicity were self-reported by parents/guardians using fixed categories (Chinese ethnicity for all participants, as inclusion was limited to Chinese children); this was collected to describe the homogeneous study population. Sex was reported by parents/guardians based on birth records.

Screenings were conducted each year from September to December for students in grades 1 to 12. Within each school, annual assessments were scheduled within a 15-day window to minimize bias from irregular follow-up intervals. The COVID-19 pandemic disrupted screening in 2020 and 2022, resulting in partial data for those years. Annual participation counts were 12,638 (2018), 13,583 (2019), 5,466 (2020), 16,026 (2021), 5,438 (2022), 11,591 (2023), and 21,559 (2024). All included participants had complete baseline and first annual follow-up measurements, as these were required to calculate first-year ΔAL. Subsequent annual follow-up was not required to be contiguous. Participants who missed one or more intermediate screening visits could contribute data again when they returned for later assessments, and no imputation was performed for unobserved visits. Thus, the pandemic-related disruptions primarily resulted in intermittent gaps in longitudinal follow-up rather than missing baseline predictor data.

This study included Chinese children aged 6–16 years at baseline who had at least 3 screening visits, including baseline, the first annual follow-up, and at least 1 additional follow-up visit, enabling longitudinal analysis. Myopia-control treatment status at baseline was ascertained from parent-reported questionnaires. Children who were already receiving myopia-control treatment at baseline, such as orthokeratology or atropine, were excluded. Initiation of myopia-control treatment during subsequent follow-up was not an exclusion criterion. Other exclusion criteria included ocular comorbidities other than myopia (e.g., amblyopia or strabismus), relevant systemic conditions, high myopia (AL ≥26.0 mm) at baseline or the first follow-up, missing essential baseline information, and physiologically implausible AL values or changes (e.g., ΔAL < −0.10 mm or >1.00 mm). A total of 7,052 participants met the eligibility criteria (Figure 1).

**Figure 1:**
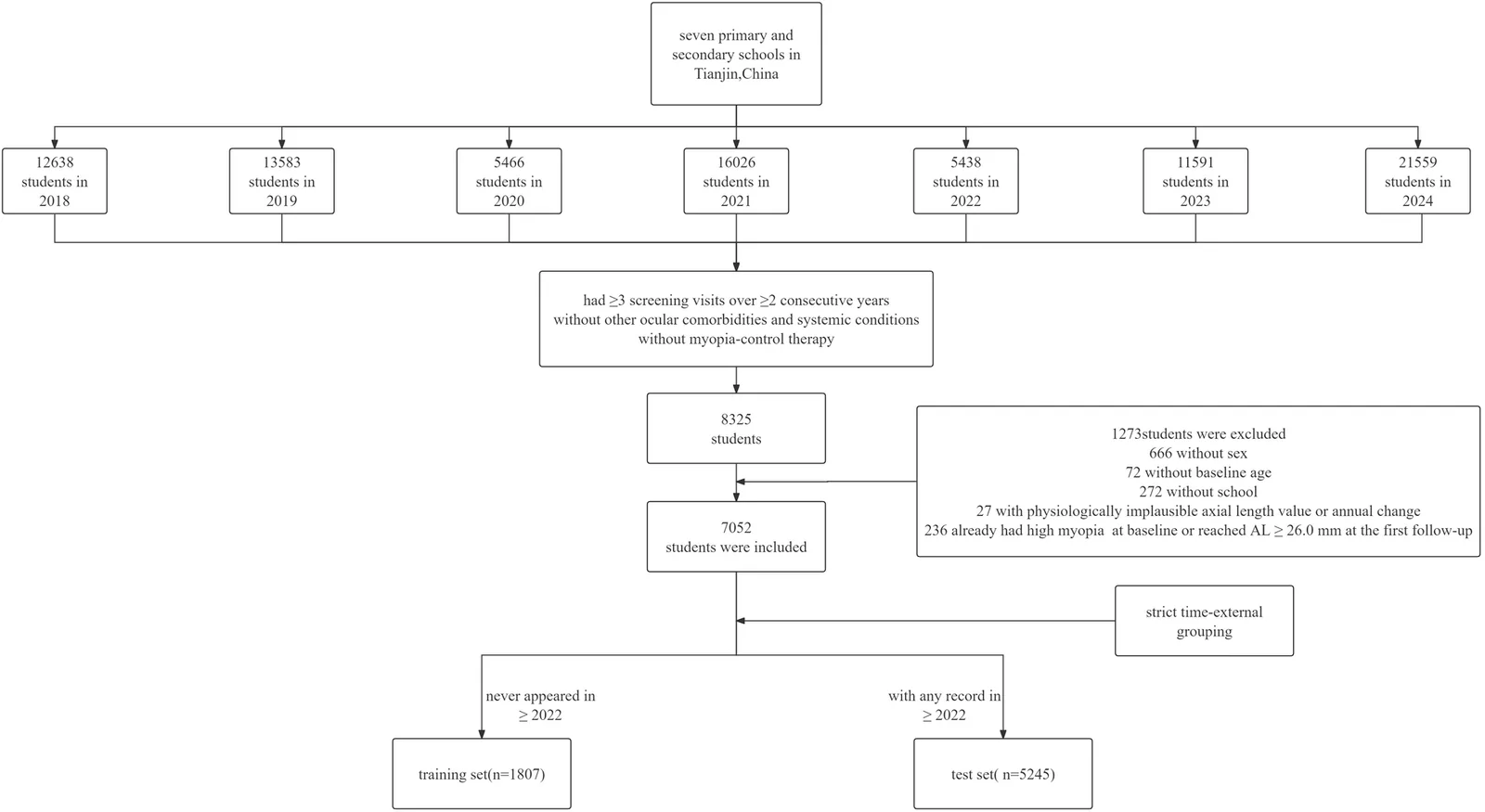
Participant flow diagram.

### Measurements

Vision screenings were performed by trained optometrists and ophthalmologists. Axial length (AL), K1 (flatter meridian of cornea), and K2 (steeper meridian of cornea) were measured using a Lenstar LS 900 biometer (Haag-Streit), with 3 consecutive recordings averaged for accuracy.

### Definition

$CR=337.5/K_{\mathrm{mean}},\;K_{\mathrm{mean}}=(K1+K2)/2\;\left(\mathrm{diopters}\right).$ The first-year axial elongation (ΔAL) was calculated as the change in AL from baseline to the first annual follow-up. Annual assessments within each school were scheduled at approximately 1-year intervals, with screening dates maintained within a 15-day window. Therefore, ΔAL was entered in millimeters without further annualization or age- or sex-specific standardization. Baseline age and start age (s) refer to age at the initial screening visit. The first annual follow-up, approximately 1 year later, served as the landmark visit at which ΔAL became available. The axial length to corneal radius ratio (AL/CR) summarizes the balance between globe length and corneal curvature and correlates strongly with refraction. AL/CR was computed as AL (mm) divided by corneal radius (32).

### Model specification and participant-level temporal cohort split

Because ΔAL becomes available only at the landmark visit, the AL + ΔAL model was formulated as a landmark prediction conditional on remaining free of high myopia at that visit. Participants with AL ≥26.0 mm at baseline or the landmark visit were excluded. We modeled subsequent high-myopia onset using discrete-time (pooled-logistic) survival with age as the time scale (33). Annual person-age rows were constructed from the landmark age (approximately s + 1) up to age 18 or event/censoring, and the baseline hazard was modeled using a natural cubic spline in age with 4 degrees of freedom to flexibly capture nonlinear age effects. Participants who did not meet the high-myopia endpoint during observed follow-up were right-censored at their last available screening. An isolated missed intermediate visit did not itself result in permanent censoring if the participant subsequently returned for screening. At age a, the conditional hazard was estimated via logistic regression with a flexible baseline in age. Cumulative risk to any target age A was computed as:

$$\hat{P}(\leq A|s)=1-\overset{A}{\underset{a=s+1}{\prod }}\;(1-{\hat{\;p}}_{a}),$$

where ${\hat{\;p}}_{a}$ is the model-based hazard at age a.

The endpoint was AL-defined high myopia (AL ≥26.0 mm); hereafter, “high myopia” refers to this endpoint. Event age was defined as the age at the first observed examination with AL ≥26.0 mm, without interpolation within unobserved intervals. Participants with any record in 2022 or later, including their earlier records, were assigned to the temporal holdout cohort (*n* = 5,245), whereas those never observed from 2022 onward formed the training cohort (*n* = 1,807), with no participant overlap.

### Predictor selection

We restricted predictors to routinely available measures with established associations with refractive status and progression: baseline AL, AL/CR, ΔAL, and sex (32, 34, 35). AL/CR was included because of its established association with refractive state. Because AL and AL/CR were highly correlated, they were evaluated in parallel rather than entered simultaneously. Final model selection was based on out-of-fold predictive performance, considering both iAUC and iBrier, together with model parsimony and clinical interpretability.

### School-level validation

To assess transportability across schools, we performed internal-external cross-validation by schools in the training set (36). At each iteration, 1 school was held out in full, models were refit on the remaining schools, and predictions for the held-out school were generated for target ages. Performance within the held-out school was summarized by the iAUC and iBrier with 95% confidence intervals (CIs). Schools with fewer than 50 participants were pooled into a single “Other schools” category.

### Model performance evaluation and calibration

Model performance was evaluated in the temporal holdout test set by computing, for each subject and target age, the cumulative risk to that age. Results are reported for ages 9–18, as incident high myopia was rare below age 9. At each target age A from 9 to 18 years, discrimination and error were quantified by inverse-probability-of-censoring weighted (IPCW) AUC and IPCW Brier score computed on the temporal holdout test set (37, 38). IPCW was used to account for right censoring when estimating age-specific AUC and Brier scores; it was not used to impute unobserved measurements during missed screening visits. The integrated AUC (iAUC) and integrated Brier (iBrier) summarized mean performance across ages 9–18. Calibration was assessed at each age using decile-based plots and two-parameter logistic recalibration. Age-specific recalibration intercepts and slopes were estimated from apparent predictions in the full training cohort and applied unchanged to the temporal holdout cohort. The reported holdout calibration plots and slopes were based on these recalibrated predictions.

Uncertainty was quantified with subject-level bootstrap (B = 1,000) to obtain 95% confidence intervals (CIs).

### Clinical application

Decision-curve analysis was performed on the temporal holdout test set to evaluate clinical utility by estimating net benefit across threshold probabilities for risk to age 18 (39). Based on the finalized, recalibrated models, we deployed an online calculator that can provide the risk of developing high myopia (https://lh1999.shinyapps.io/High-Myopia-Risk-Calculator/). In the calculator, “Start age (s)” corresponds to age at the baseline screening visit, whereas risk prediction for the AL + ΔAL model is conditional on remaining free of high myopia at the first annual follow-up, after ΔAL has become available. “Prediction age (A)” denotes the target age to which cumulative risk is estimated. The calculator provides an accessible interface for displaying model-estimated cumulative risks.

### Data analysis

Model performance was evaluated in the temporal holdout test set by computing, for each subject and target age, the cumulative risk to that age. Results are reported for ages 9–18, as incident high myopia was rare below age 9. Only the left eye was analyzed. Continuous variables are reported as mean (SD) or median (IQR), and categorical variables as number (percentage). Normality was checked with the Shapiro–Wilk test. Differences between the training and temporal holdout cohorts were examined with standardized mean differences (SMDs). For prediction performance, we summarized AUC and Brier score at ages 9–18 and reported their across-age means (iAUC, iBrier) with 95% bootstrap confidence intervals. All analyses were performed in R (version 4.5.1, R Foundation for Statistical Computing); |SMD| < 0.10 was considered negligible, 0.10–0.20 small, 0.20–0.50 moderate, and >0.50 large. Two-sided *p* values < 0.05 were considered statistically significant.

## Results

### Baseline characteristics

Table 1 summarizes the characteristics of the total 7,052 children (training = 1,807; temporal holdout test = 5,245). At baseline, the cohort was young [overall median age 9 years (IQR 7–10)] and 43.6% were female. AL at baseline averaged 23.38 ± 1.01 mm overall and was slightly shorter in the test set than in the training set (23.28 ± 0.96 vs. 23.67 ± 1.08 mm; SMD = 0.384). The AL/CR ratio was similar across sets [overall median 2.98 [2.92–3.07]; train 2.97 [2.91–3.06] vs. test 2.99 [2.92–3.08]; SMD = −0.125]. ΔAL was modestly greater in the test set [0.29 mm (0.18–0.44)] than in the training set [0.23 mm (0.13–0.37); SMD = −0.325]. The proportion of participants who developed high myopia (AL ≥ 26.0 mm) was 8.2% overall (5.0% in the training cohort vs. 9.3% in the temporal holdout cohort; SMD = −0.167). Female proportion was nearly identical (45.0% in train vs. 43.2% in test; SMD = 0.038). These differences indicate a shift in baseline case mix between the training and temporal holdout cohorts, particularly with respect to baseline AL and early axial elongation.

**Table 1:** Participant characteristics and SMDs comparing training and temporal holdout cohorts.

Characteristic	Overall (*N* = 7,052)	Train (*N* = 1,807)	Test(*N* = 5,245)	SMD (Train vs. Test)
Age at baseline (years)	9 [7, 10]	10 [7, 10]	8 [7, 10]	0.773
Female, *n* (%)	3,078（43.6%）	814（45%）	2,264（43.2%）	0.038
Axial length at baseline (mm)	23.38 ± 1.01	23.67 ± 1.08	23.28 ± 0.96	0.384
AL/CR ratio at baseline	2.98 [2.92, 3.07]	2.97 [2.91, 3.06]	2.99 [2.92, 3.08]	−0.125
The first year axial elongation, (mm)	0.28 [0.17, 0.43]	0.23 [0.13, 0.37]	0.29 [0.18, 0.44]	−0.325
High myopia, *n* (%)	577（8.2%）	90（5.0%）	487（9.3%）	−0.167

(AL, axial length; ΔAL, first-year axial elongation; AL/CR, axial length to corneal radius ratio; SMD, standardized mean difference).

### Comparative performance of two parallel models

Table 2 shows model performance and predictor odds ratios from the discrete-time pooled-logistic models in the training cohort. AL-based specifications outperformed AL/CR counterparts within both frameworks. Without ΔAL, AL achieved iAUC 0.94 (95% CI 0.92–0.96) with iBrier 0.11 (95% CI 0.09–0.14), whereas AL/CR reached iAUC 0.81 (95% CI 0.77–0.87) with a higher iBrier 0.17 (95% CI 0.15–0.19). With ΔAL included, AL + ΔAL yielded iAUC 0.97 (95% CI 0.95–0.98) and iBrier 0.07 (95% CI 0.06–0.10), while AL/CR + ΔAL produced iAUC 0.83 (95% CI 0.78–0.86) and iBrier 0.17 (95% CI 0.15–0.19). Given their consistently higher discrimination and lower prediction error, AL and AL + ΔAL were retained as the final models for subsequent temporal holdout evaluation, calibration, and clinical application.

**Table 2:** The performance of two parallel models in the training cohort.

Predictors	AL (M1,iAUC=0.94,iBrier=0.11）		AL/CR (M2,iAUC=0.81,iBrier=0.17）		AL + ΔAL (M3,iAUC=0.97,iBrier=0.07）		AL/CR + ΔAL (M4,iAUC=0.83,iBrier=0.17）	
	OR (95% CI)	*P* value	OR (95% CI)	*P* value	OR (95% CI)	*P* value	OR (95% CI)	*P* value
AL (per 0.1 mm)	1.34（1.27–1.42）	＜0.001	-		1.58（1.45–1.73）	＜0.001	-	-
ΔAL (per 0.1 mm)	-	-	-		2.45（2.02–3.00）	＜0.001	2.46（2.02–3.00）	＜0.001
AL/CR (per 0.01)	-	-	1.00（0.98–1.01）	0.92	-	-	1.00（0.99–1.02）	0.54
Male	0.95（0.60–1.52）	0.84	0.95（0.60–1.52）	0.84	0.96（0.58–1.56）	0.87	0.96（0.58–1.56）	0.83

All models included a natural cubic spline for age with 4 degrees of freedom.

AL, baseline axial length; ΔAL, the first-year axial elongation; AL/CR, axial length to corneal radius ratio.

### School-level internal-external validation

Across the leave-one-school-out analyses, both models showed consistent performance across the participating schools (Figure 2). The AL + ΔAL model showed uniformly higher discrimination and lower prediction error than AL in every held-out school. School-specific mean iAUC for AL + ΔAL ranged from 0.92 to 0.97, with iBrier values ranging from 0.02 to 0.19 (Figure 2B). For the AL model, school-specific iAUC ranged from 0.81 to 0.97, with iBrier values ranging from 0.03 to 0.26 (Figure 2A). Taken together, these findings indicate relatively stable performance across the participating schools within this cohort.

**Figure 2:**
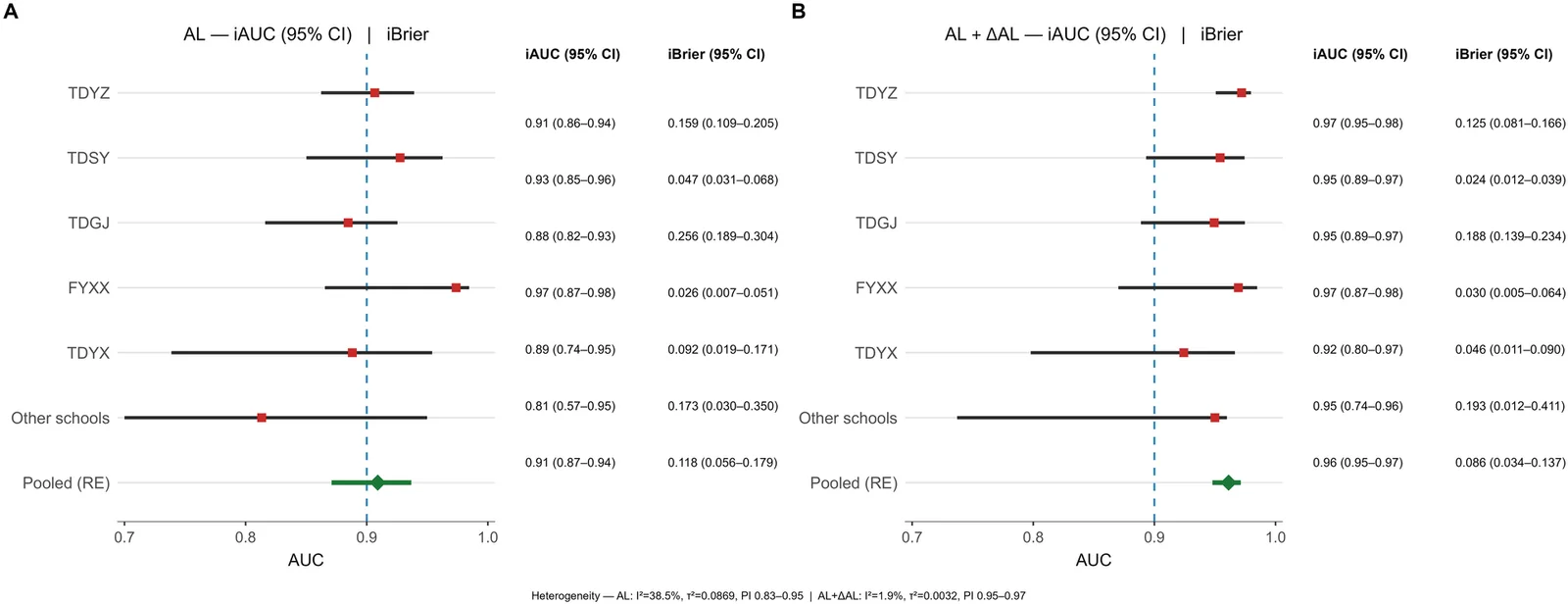
School-level internal-external validation performance in the training cohort. **(A)** Axial length + sex model. **(B)** Axial length + ΔAL model. Forest-plot summaries of the age-integrated area under the receiver operating characteristic curve (iAUC) and age-integrated Brier score (iBrier) by school.

### Predictive discrimination and calibration

In the temporal holdout cohort, both models showed strong discrimination across ages 9–18, with the AL + ΔAL model consistently outperforming the AL model (Table 3 and Figure 3). The age-averaged AUC (iAUC) was 0.851 (95% CI 0.833–0.869) for AL and 0.919 (95% CI, 0.902–0.933) for AL + ΔAL (Table 3 and Figure 3A). Concordantly, overall prediction error was lower for AL + ΔAL: the age-averaged Brier score (iBrier) was 0.074 (95% CI, 0.068–0.081) versus 0.087 (95% CI, 0.080–0.095) for AL (Table 3 and Figure 3B). As expected, AUC declined with age (AL: 0.96 at age 9–0.84 at age 18; AL + ΔAL: 0.98–0.91), while the Brier score increased with age, peaking around age 17 (0.20 for AL; 0.17 for AL + ΔAL) and tapering at age 18 (0.14 and 0.12, respectively).

**Table 3:** Predictive performance of axial length and axial length + ΔAL models from ages 9 to 18 years.

Age (years)	Model AL		Model AL + ΔAL		High-myopia cases, *n*
	AUC (95% CI)	Brier score (95% CI)	AUC (95% CI)	Brier score (95% CI)	
9	0.961 (0.944–0.976)	0.004 (0.002–0.007)	0.985 (0.975–0.992)	0.004 (0.002–0.006)	13
10	0.953 (0.938–0.968)	0.009 (0.006–0.012)	0.979 (0.963–0.990)	0.007 (0.005–0.009)	35
11	0.931 (0.908–0.949)	0.018 (0.014–0.022)	0.960 (0.940–0.976)	0.014 (0.012–0.017)	90
12	0.904 (0.884–0.922)	0.036 (0.031–0.042)	0.934 (0.914–0.952)	0.030 (0.026–0.034)	178
13	0.875 (0.853–0.896)	0.054 (0.048–0.060)	0.922 (0.903–0.939)	0.046 (0.041–0.052)	267
14	0.865 (0.844–0.885)	0.078 (0.070–0.086)	0.922 (0.905–0.937)	0.066 (0.059–0.073)	351
15	0.844 (0.825–0.864)	0.117 (0.106–0.128)	0.914 (0.897–0.931)	0.099 (0.089–0.108)	422
16	0.831 (0.805–0.854)	0.142 (0.128–0.156)	0.911 (0.890–0.930)	0.118 (0.106–0.130)	461
17	0.816 (0.785–0.845)	0.201 (0.178–0.225)	0.911 (0.882–0.935)	0.173 (0.151–0.195)	477
18	0.843 (0.808–0.878)	0.140 (0.120–0.161)	0.915 (0.877–0.945)	0.123 (0.104–0.143)	487
Integrated	0.851 (0.833–0.869)	0.087 (0.080–0.095)	0.919 (0.902–0.933)	0.074 (0.068–0.081)	

**Figure 3:**
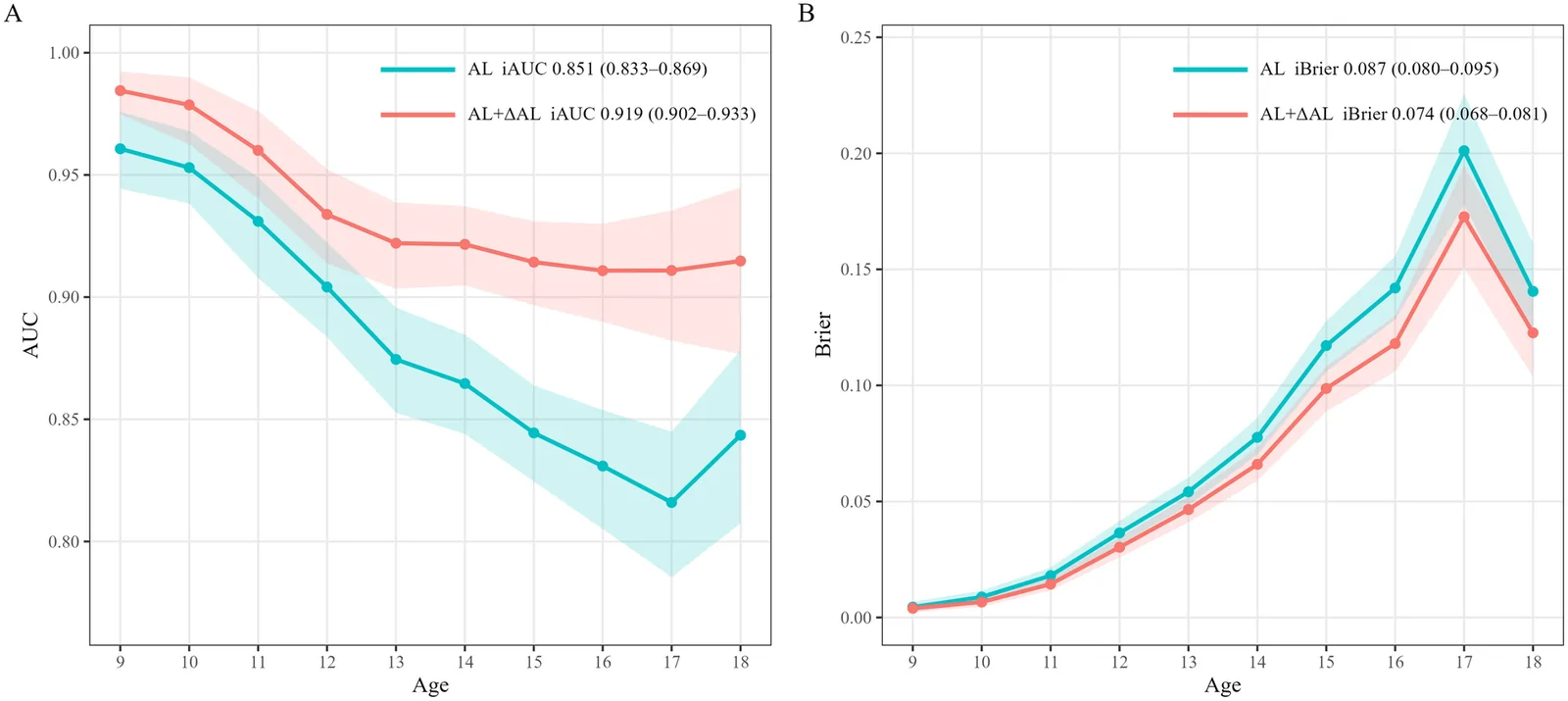
Predictive performance of axial length and axial length + ΔAL models From ages 9 to 18 years. **(A)** Age-specific discrimination (AUC) of the AL and AL + ΔAL models from ages 9 to 18. **(B)** Age-specific prediction error (Brier score) of the AL and AL + ΔAL models from ages 9 to 18. Shaded ribbons indicate 95% confidence intervals.

After application of the training-derived recalibration parameters, calibration in the temporal holdout cohort was acceptable for both models across all ages (Figure 4). Decile-based calibration plots of the recalibrated predictions closely tracked the 45° line, and the corresponding calibration slopes were near 1.0 across ages 9–18 (AL: *β*=1.00–1.03; AL + ΔAL: *β*=1.00–1.02). Small deviations and wider intervals at older ages likely reflected fewer events, but no systematic over- or under-prediction was evident. Overall, both models showed strong discrimination, low prediction error, and good calibration from ages 9–18, with consistently better performance for AL + ΔAL.

**Figure 4:**
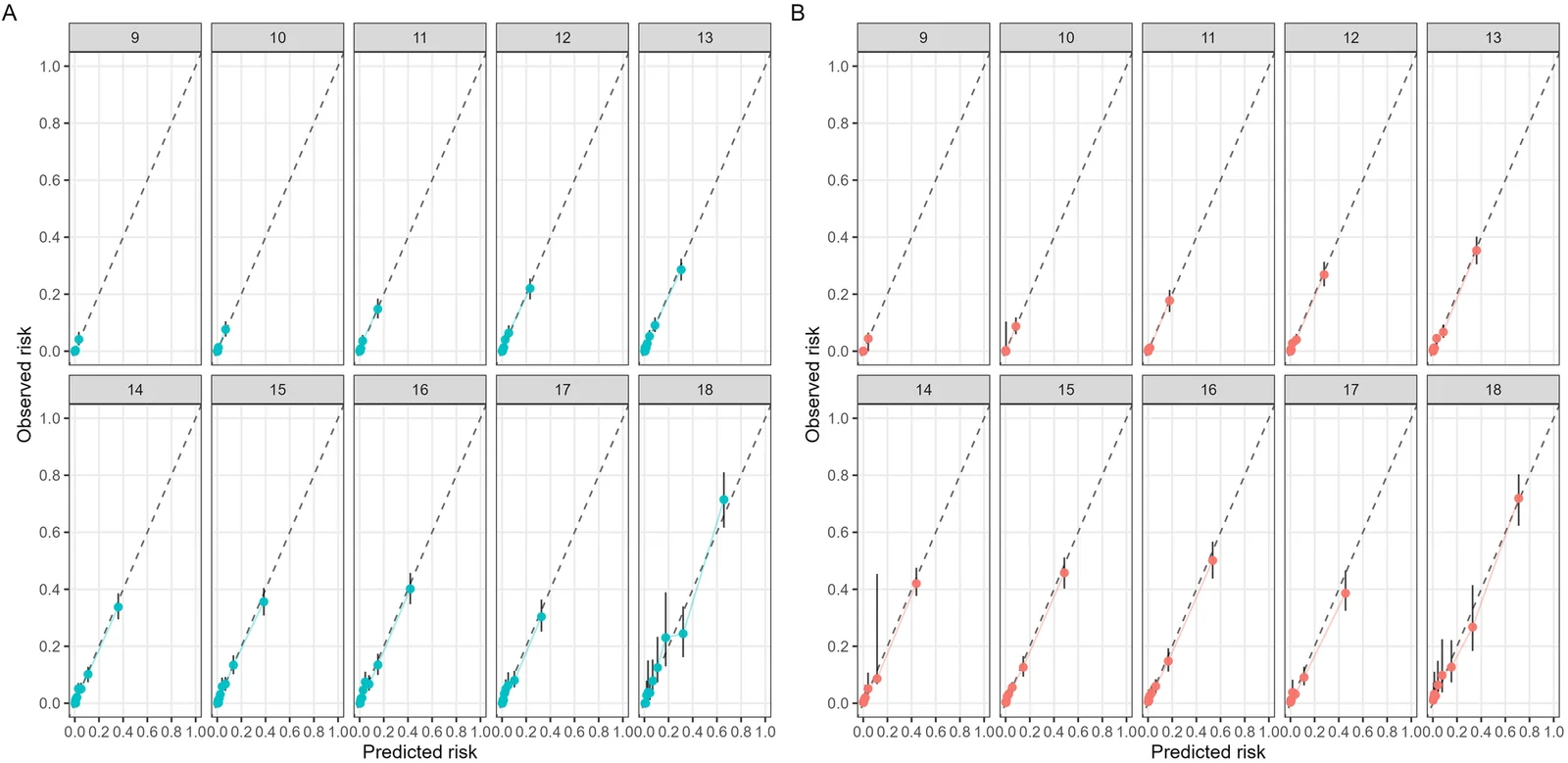
Decile-based calibration plots of axial length and axial length + ΔAL models from ages 9 to 18 years. **(A)** Axial length + sex model. **(B)** Axial length + ΔAL model. For each age, points represent grouped predictions (mean predicted vs. observed risk). Dashed line indicates perfect calibration (45°).

### Practical implementation of risk estimates

Decision-curve analysis in the temporal holdout cohort showed net benefit across thresholds of 0.05–0.85, with higher net benefit for AL + ΔAL than for AL (Figure 5C). Both models outperformed treat-all and treat-none strategies over most of this range. To facilitate interpretation of the model estimates, we provided sex-stratified cumulative-risk tables, age-indexed risk curves (Figure 5A), risk estimates according to first-year axial elongation (Figure 5B), and an online calculator for individualized risk estimation (Figure 5D).

**Figure 5:**
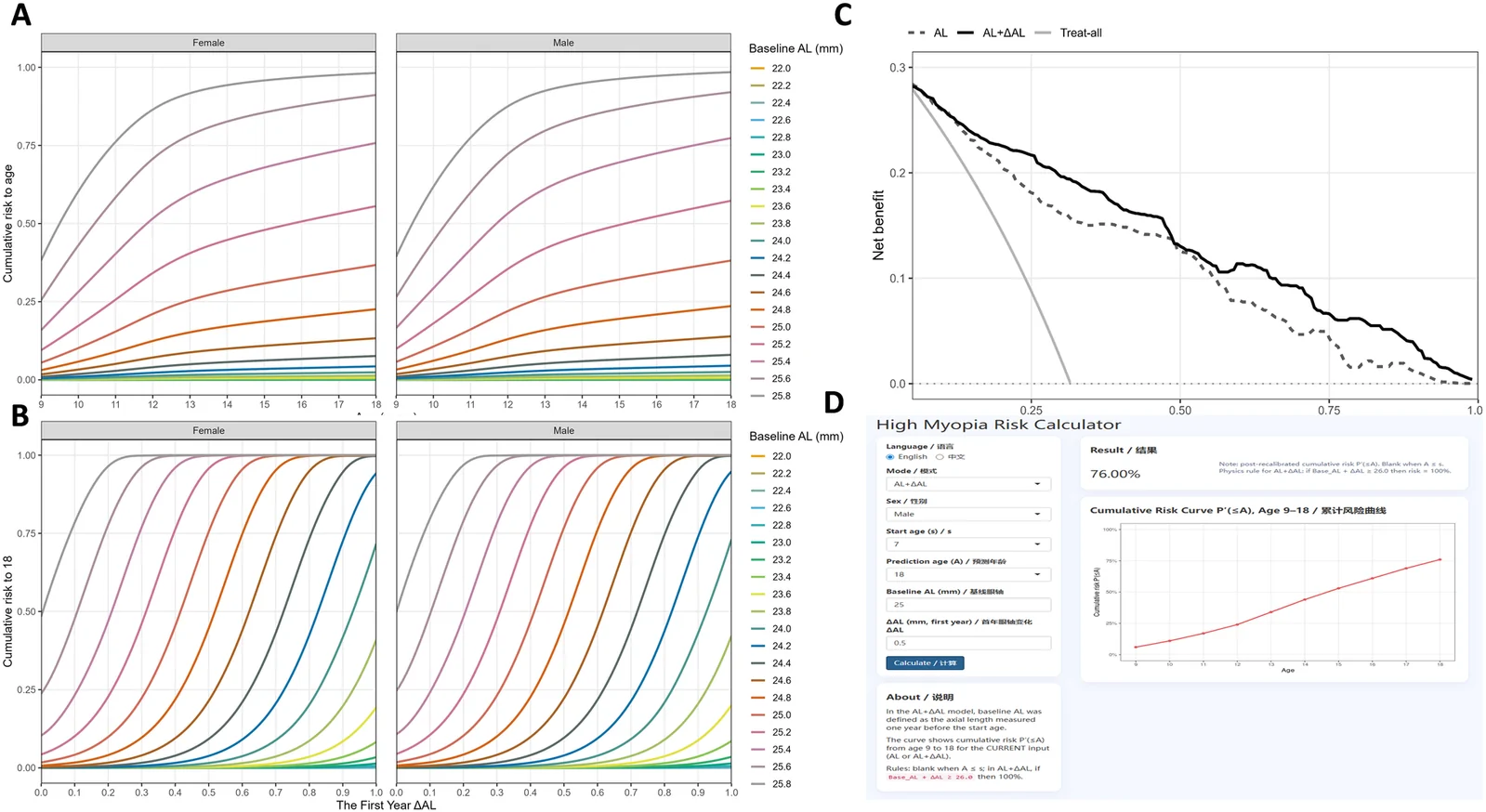
Reference curves and clinical utility of axial length and axial length + ΔAL models. **(A)** Age-indexed cumulative-risk curves from ages 9 to 18 for the AL model, stratified by baseline axial length and sex (axial length model). **(B)** Cumulative risk to age 18 estimated by the AL + ΔAL model for a child aged 7 years at baseline, plotted against first-year ΔAL and stratified by baseline axial length and sex. **(C)** Decision-curve analysis (net benefit across thresholds). **(D)** Bilingual web-based high-myopia risk calculator.

## Discussion

In this 7-year school-based longitudinal cohort study, we developed and validated 2 pragmatic prediction models for estimating the age-indexed cumulative risk of high myopia (defined as AL ≥26.0 mm) in Chinese children aged 6 to 16 years at baseline. The model incorporating both baseline AL and first-year ΔAL demonstrated superior discrimination compared with the AL-only model (iAUC, 0.919 vs. 0.851), highlighting the added prognostic value of early axial elongation as a dynamic marker of progression risk. These findings underscore the potential of routine, non-invasive biometry measures to facilitate individualized risk stratification, enabling targeted myopia-control interventions to mitigate the long-term burden of high myopia and its associated complications, such as myopic maculopathy and retinal detachment (3, 4, 7–10, 40, 41).

AL/CR remains a clinically meaningful biometric measure. Previous studies have demonstrated a strong association between AL/CR and refractive state, and higher AL/CR values have been reported in children with high compared with low myopia (32, 42). However, AL/CR may be less suitable as a longitudinal marker of myopia progression, as the relationship between changes in AL/CR and refractive progression differs according to the degree of myopia (42). In our cohort, the AL-based specifications substantially outperformed the corresponding AL/CR-based models in both discrimination and prediction error. This difference may partly reflect the structural definition of the study endpoint. Because high myopia was defined as AL ≥26.0 mm, baseline AL directly captures proximity to the event threshold, whereas AL/CR additionally incorporates corneal curvature and more broadly reflects refractive status.

Machine-learning methods have been used to predict spherical equivalent refraction and AL in children, demonstrating the potential of longitudinal ocular biometry for data-driven risk estimation (43). However, prior studies have often relied on spherical equivalent refraction or more complex inputs, including fundus imaging, genetic data, and longitudinal electronic health records, which may limit their accessibility in routine school screening or outpatient settings (27–31, 44). For example, a time-aware deep-learning model has shown promise in predicting spherical equivalent up to 7 years ahead from irregular longitudinal clinical records, although such approaches require sufficiently rich historical data and computational infrastructure (44). Our study addresses a different implementation goal. Rather than developing a machine-learning or deep-learning algorithm, we used a conventional, interpretable statistical prediction framework based on routinely available biometric measures. With only baseline AL and early ΔAL as the principal biometric predictors, the model achieved high discrimination while providing directly interpretable age-specific risk estimates, reference tables, and an online calculator. This aligns with the 2025 International Myopia Institute (IMI) digest, which emphasizes personalized monitoring of axial elongation to guide myopia-control interventions (45).

These findings may help inform age-specific risk stratification. By estimating cumulative risk from ages 9–18, these models can inform decision-making for myopia-control strategies, such as orthokeratology or low-dose atropine, particularly in high-risk children where early intervention yields the greatest axial length reduction (12–16). Decision-curve analysis showed net benefit across the evaluated threshold range. In school-based programs, these models may help prioritize follow-up for children at higher risk. Performance was stable across the participating schools, but broader application requires independent external validation.

Study strengths include the large school-based longitudinal cohort, complete participant separation between the development and temporal holdout cohorts, school-level internal-external validation, and the use of routinely available predictors.

However, several limitations should be considered. First, all participants were Chinese children from Tianjin. Therefore, the school-level internal-external validation reflects transportability within this regional cohort rather than generalizability to other geographic or ethnic populations. Independent external validation and, where necessary, recalibration are required before broader use.

Second, COVID-19 disruptions resulted in intermittent and potentially non-random follow-up. Because the temporal holdout partly depended on continued observation into 2022 or later, differences between the training and holdout cohorts may reflect temporal changes, differential follow-up, or both. Intermittent gaps between observed examinations also introduced uncertainty in the exact timing of high-myopia onset. In addition, myopia-control treatment initiated during follow-up was not used for censoring; therefore, the observed risk trajectories do not represent a strictly untreated natural course. First-year ΔAL was not age- or sex-standardized, and possible age-related differences in its prognostic effect were not explicitly modeled. Finally, AL ≥26.0 mm is a structural definition of high myopia and is not equivalent to refractive high myopia at the individual level.

In summary, these models provide an interpretable framework for estimating age-indexed risk of AL-defined high myopia using routinely available axial biometry. Future research should focus on multi-regional and prospective external validation, refinement of clinically meaningful risk thresholds, and evaluation of the models within real-world school and outpatient screening pathways. By enabling earlier identification of children at elevated risk, this approach may contribute to more timely and proportionate myopia-control management.

## Data Availability

Data are available upon reasonable request. The full study protocol and study data can be obtained upon request from the corresponding author. The data cannot be shared publicly due to regulations of the local ethics committee. Data may be made available to researchers who meet the criteria for access to confidential data and upon institutional review board approval.
